# EGFR-Targeted Photodynamic Treatment of Triple Negative Breast Cancer Cell Lines Using Porphyrin–Peptide Conjugates: Synthesis and Mechanistic Insight

**DOI:** 10.3390/molecules30173533

**Published:** 2025-08-29

**Authors:** Miryam Chiara Malacarne, Federica Randisi, Emanuela Marras, Stefano Giovannardi, Paolo Dognini, Alan Mark Simm, Francesca Giuntini, Marzia Bruna Gariboldi, Enrico Caruso

**Affiliations:** 1 Department of Biotechnology and Life Sciences (DBSV), University of Insubria, Via J. H. Dunant 3, 21100 Varese, Italy; mc.malacarne1@uninsubria.it (M.C.M.); frandisi1@studenti.uninsubria.it (F.R.); emanuela.marras@uninsubria.it (E.M.); stefano.giovannardi@uninsubria.it (S.G.); enrico.caruso@uninsubria.it (E.C.); 2 Centre for Neuroscience, Department of Biotechnology and Life Sciences (DBSV), University of Insubria, Via J. H. Dunant 3, 21100 Varese, Italy; 3 School of Pharmacy and Biomolecular Sciences, Byrom Street Campus, Liverpool John Moores University, Liverpool L3 3AF, UK; dogninip@gmail.com (P.D.); f.giuntini@ljmu.ac.uk (F.G.); 4 Faculty of Sciences, Byrom Street Campus, Liverpool John Moores University, Liverpool L3 3AF, UK; a.m.simm@ljmu.ac.uk

**Keywords:** breast cancer, triple-negative breast cancer (TNBC), photodynamic therapy (PDT), diarylporphyrins, therapeutic peptide, epidermal growth factor receptor (EGFR), tumor targeting

## Abstract

Triple-negative breast cancer (TNBC) is an aggressive subtype of breast cancer characterized by the absence of estrogen receptor, progesterone receptor, and human epidermal growth factor receptor 2, limiting the efficacy of conventional targeted therapies. As a result, novel therapeutic strategies are urgently needed. Photodynamic therapy (PDT), which relies on the activation of photosensitizers (PSs) by light to induce cytotoxic effects, has emerged as a promising alternative for TNBC treatment. Furthermore, the conjugation of PSs with targeting peptides has demonstrated enhanced selectivity and therapeutic efficacy, particularly for porphyrin-based photosensitizers. In this study, we report the synthesis of novel porphyrin–peptide conjugates designed to selectively target the epidermal growth factor receptor (EGFR), which is frequently overexpressed in TNBC. The conjugates were prepared via thiol displacement of the meso-nitro group in a 5,15-diarylporphyrin scaffold using EGFR-binding peptides. Photodynamic activity was evaluated in two EGFR-overexpressing TNBC cell lines. Cellular uptake of the conjugates correlated with EGFR expression levels, and PDT treatment resulted in differential induction of necrosis, apoptosis, and autophagy. Notably, the conjugates significantly inhibited EGFR-expressing cell line migration, a critical hallmark of metastatic progression. These findings underscore the potential of EGFR-targeted porphyrin–peptide conjugates as promising PDT agents for the treatment of TNBC.

## 1. Introduction

Cancer is a major global health challenge and currently the second leading cause of death worldwide following cardiovascular diseases [1,2]. Among all cancers, breast cancer (BC) is the most commonly diagnosed malignancy and cause of cancer-related mortality in women, accounting for approximately 33% of female cancers. Alarmingly, the global incidence of BC continues to rise at a rate of ca. 0.5% per year [3,4]. A particularly aggressive and therapeutically challenging subtype of BC is triple-negative breast cancer (TNBC), which constitutes 10–15% of all breast cancer cases and is defined by the absence of estrogen receptor α (ERα), progesterone receptor (PR), and human epidermal growth factor receptor 2 (HER2) expression [5,6]. TNBC is associated with poor prognosis due to its high metastatic potential, early recurrence, and limited response to conventional therapies. Currently, systemic chemotherapy remains the mainstay of treatment; however, it is often accompanied by significant side effects and fails to prevent relapse and metastatic progression [7,8,9]. This pressing clinical gap highlights the urgent need for innovative and more effective treatment strategies for TNBC [10,11]. Among emerging alternatives, photodynamic therapy (PDT) has gained increasing attention as a promising and highly selective therapeutic modality. PDT is based on the activation of a photosensitizer (PS) by visible light in the presence of molecular oxygen, leading to the generation of reactive oxygen species (ROS), particularly cytotoxic singlet oxygen (^1^O_2_), which induces targeted tumor cell death [12,13,14].

A key advantage of PDT is its inherent two-fold selectivity arising from both the preferential accumulation of PSs in diseased tissues and the spatially confined activation of these agents through targeted light irradiation. This dual mechanism confers a minimally invasive profile to PDT, making it particularly attractive for oncological applications [15,16]. Over the years, a wide array of PSs has been developed, with several already approved for clinical use and many others under active investigation in both preclinical and clinical studies, including those focused on breast cancer [15,17,18]. Despite its promising therapeutic potential, the broader clinical adoption of PDT remains limited by several challenges associated with PSs, most notably, dark toxicity, inadequate tumor selectivity, and suboptimal biocompatibility [19]. Addressing these limitations is crucial for enhancing the safety and efficacy of PDT, particularly in the treatment of aggressive and refractory cancers such as triple-negative breast cancer. To overcome these issues and enhance the specificity and accumulation of PSs in target tissues, various strategies have been employed, particularly the conjugation of PSs with polymers, nanoparticles, liposomes, monoclonal antibodies, antibody fragments, peptides or proteins (e.g., transferrin, epidermal growth factor—EGF, and insulin), carbohydrates, somatostatin, folic acid, and other targeting moieties [20,21]. The epidermal growth factor receptor (EGFR), a transmembrane receptor tyrosine kinase that plays a central role in regulating various cellular processes, including survival, proliferation, and differentiation [22,23], has recently emerged as a promising target for tumor-specific delivery of photosensitizers, and several EGFR-targeting strategies, ranging from the use of monoclonal antibodies and EGF conjugates to small-molecule inhibitors and nanocarrier-based systems, have demonstrated encouraging results in enhancing cellular uptake, selectivity, and photodynamic efficacy [24,25,26]. Recent studies have reported EGFR overexpression in TNBC patients, with prevalence rates ranging from 13% to 89%, depending on the cohort and detection methodology [27]. The conjugation of porphyrin-based PSs with peptides has attracted considerable attention as an approach to enhance the selective delivery of PSs to tumor cells through receptor-mediated mechanisms, and to mitigate unfavorable physicochemical properties (e.g., low aqueous solubility, tendency to aggregation, etc.), thereby improving the overall pharmacokinetic and pharmacodynamic profiles of the photosensitizers [28,29,30].

Tumor-Targeting Peptides (TTPs) have been studied as targeting agents to deliver drugs to cancer cells due to their ability to bind tumor-specific cell markers. Once bound to the target cells, TTPs are internalized through active transport mechanisms such as endocytosis [30,31,32,33]. Examples of TPPs that were developed to specifically target EGFR were previously reported; these species were shown to facilitate enhanced tumor selectivity and intratumoral delivery of therapeutic agents in EGFR-overexpressing malignancies [34,35,36].

The targeted delivery of BODIPY-based photosensitizers to TNBC cells has been successfully explored using EGFR-binding peptides, as demonstrated by Zhao and colleagues in 2017. In their study, EGFR-targeting peptide sequences were conjugated to BODIPY fluorophores via a polyethylene glycol (PEG) linker to enhance cellular uptake. Fluorescence imaging revealed that these peptide-BODIPY conjugates preferentially accumulated in the cytoplasm of EGFR-overexpressing TNBC cells, highlighting their potential for selective photodynamic therapy [37]. Furthermore, the synthesis and activity of peptide-decorated nano-photosensitizing systems with porphyrins, phthalocyanines, and chlorin e6 have also been explored for EGFR-targeted PDT [38,39,40,41].

Efforts have also focused on conjugating EGFR-binding peptides to the meso-aryl substituents of the porphyrin core to enhance tumor selectivity and cellular uptake [41,42]. However, to the best of our knowledge, no strategy has been reported to date that enables the direct conjugation of a fully deprotected peptide to the meso-position of a porphyrin scaffold. This represents an unmet challenge and a potential opportunity for developing novel peptide–porphyrin conjugates with lower molecular weight and improved biological performance.

Previous studies conducted by our research groups demonstrated the promising potential of 5,15-meso-diarylporphyrins as photosensitizers (PSs) for anticancer photodynamic therapy [43,44,45], and have focused on optimizing a chemoselective ligation strategy for the conjugation of porphyrins to fully deprotected peptides in solution exploiting the nucleophilic aromatic substitution of activated aryl halides or nitro groups by thiols [46,47,48]. Given that aromatic nitro groups are susceptible to thiol-mediated displacement, we hypothesized that meso-nitrated 5,15-diarylporphyrins could serve as suitable electrophilic platforms for covalent attachment of cysteine-containing peptides. Accordingly, we designed TNBC-targeting porphyrin–peptide conjugates by anchoring epidermal growth factor receptor (EGFR)-binding peptide sequences, derived from a segment of human EGF (see below for sequence selection criteria), to the porphyrin scaffold. Each 12-mer peptide was functionalized with an N-terminal cysteine residue to enable site-specific conjugation at the porphyrin meso-position. The resulting conjugates were synthesized, fully characterized, and evaluated for their photodynamic efficacy in triple-negative breast cancer (TNBC) cell models.

## 2. Results and Discussion

### 2.1. Chemistry

#### 2.1.1. Porphyrin Synthesis

The presence of two unsubstituted meso-positions in 5,15-diarylporphyrins allows for a wider range of chemical modifications compared to the corresponding tetraaryl-substituted species [41]. The selective addition of functional groups such as nitro or bromo groups at the meso-position can be achieved through straightforward synthetic methods. These modifications offer versatile reactive sites for further functionalization, a strategy that has been successfully used to create a range of diverse porphyrin derivatives.

Three principal synthetic strategies for the preparation of 5,15-diarylporphyrins are well-documented in the literature: MacDonald’s synthesis, which involves the acid-catalyzed condensation of a dicarbinol- or diformyl-dipyrromethane with dipyrromethane; Baldwin’s synthesis, in which two meso-substituted dipyrromethanes are condensed with two equivalents of an orthoester; and Lindsey’s synthesis, which entails the reaction of two dipyrromethanes with two aryl aldehydes under acidic conditions [49,50,51]. Each method proceeds via an initial acid-catalyzed condensation step, yielding a porphyrinogen intermediate, which is subsequently oxidized, typically using quinone-based oxidants, to afford the corresponding porphyrin macrocycle.

**Figure 1:**
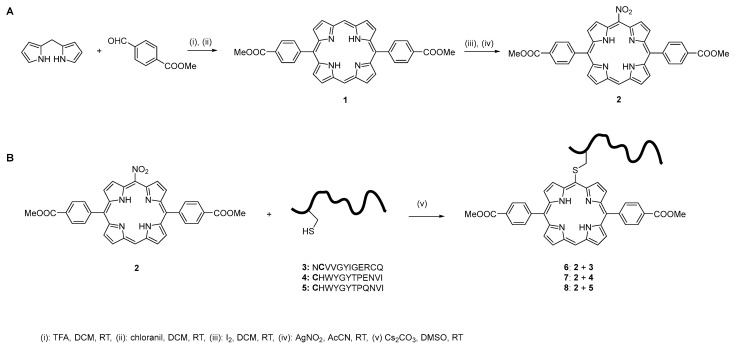
(**A**) Synthesis of compounds **1** and **2**. (**B**) General procedure used to obtain the conjugates **6**–**8**, subsequently tested on TNBC cells.

In this study, we used Lindsey’s synthetic method because of its high versatility and ability to produce structurally diverse libraries of 5,15-diarylporphyrins. Specifically, the symmetric diarylporphyrin **1** was synthesized through a mixed acid-catalyzed condensation of dipyrromethane with methyl 4-formylbenzoate, followed by oxidative cyclization to form the desired porphyrin macrocycle (Figure 1A) [49]. The porphyrinogen intermediate was then oxidized with chloranil, and the crude product was purified by column chromatography and recrystallized, resulting in the target porphyrin with a 61% yield. The choice of methyl 4-formylbenzoate affords a 5,15-diarylporphyrin that allows further potential modification, as hydrolysis of the ester function reveals two carboxylic moieties that lend themselves to further functionalization.

We recently focused on developing the conditions to perform the conjugation of a fully deprotected peptide to a porphyrin via thiol-fluoride displacement. Meso-fluorination of octaethylporphyrin has been described with the use of cesium fluoroxysulfate [52] and with fluoropyridinium triflate [53]. Our attempt to apply the method developed by Naruta et al. did not yield the desired *meso*-fluorinated species, so we turned our attention to introducing a nitro group in the target position. We reasoned that this strategy would provide a porphyrin with a suitable leaving group for thiol-led displacement and a more robust synthesis [54]. Selective nitration at one of the meso-positions was achieved through treatment with iodine and silver nitrate in an acetonitrile/dichloromethane (ACN/DCM) solvent mixture, yielding compound **2** in 85% yield, in accordance with the procedure described by Baldwin and co-workers [50]. The nitration reaction occurs rapidly at room temperature, reaching completion within a few minutes. The target compound **2** was recovered from the supernatant of the reaction mixture suspension. Mass spectrometry (MS) and proton nuclear magnetic resonance (^1^H-NMR) spectroscopy confirmed the identity and purity of the compound, indicating that it was suitable for subsequent use without needing further purification.

Recently, Hossein-Nejad-Ariani and colleagues reported the high-throughput screening of a peptide library designed to identify sequences exhibiting strong binding affinity toward EGFR in TNBC cells [55]. The parent peptide sequence was derived from a 12-amino acid segment of the native EGF ligand (NCVVGYIGERCQ, herein referred to as peptide **3**). This sequence served as the template for the rational design of a series of modified analogs aimed at preserving or enhancing binding affinity toward EGFR. Several of these engineered peptides were subsequently evaluated for their receptor-binding properties and biological activity in TNBC cell lines [37,55].

In the present study, employing a rational design strategy, we generated a series of 15 peptide sequences, each comprising 12 amino acids (Figure S1) derived from peptide **3**. A cysteine residue was appended to the N-terminus of each sequence to provide a free thiol group for site-specific conjugation to porphyrin **2** via aromatic nucleophilic substitution [46]. Sequence variants were generated through systematic permutations of amino acids, with initial modifications guided by the steric properties of the side chains. In cases where multiple substitutions were possible, the polarity of the residue was also considered. Structural analysis confirmed that substitutions at positions **aa5** and **aa6** significantly disrupted peptide folding and were therefore excluded from further design iterations [37]. In contrast, modifications at position **aa8** had minimal impact on the overall peptide conformation, provided the side chain volume remained within a similar range. The designed peptide sequences were initially subjected to conformational analysis using the online tool PEP-FOLD [56]. Sequences exhibiting structural similarity to the reference peptide **3** were subsequently evaluated via molecular docking studies to assess whether their binding mode to EGFR resembled that of peptide **3**. The docking results revealed that peptides **4** (CHWYGYTPENVI) and **5** (CHWYGYTPQNVI) adopt binding poses comparable to peptide **3**, engaging EGFR in proximity to the EGF binding pocket. Both peptides demonstrated partial spatial overlap with the EGF binding site. Notably, in both cases, the **Tyr6** residue formed a hydrogen bond with the side chain of **Arg310** on EGFR, suggesting a conserved interaction critical for receptor binding.

#### 2.1.2. Synthesis of Peptides **3**–**5**

**Figure 2:**
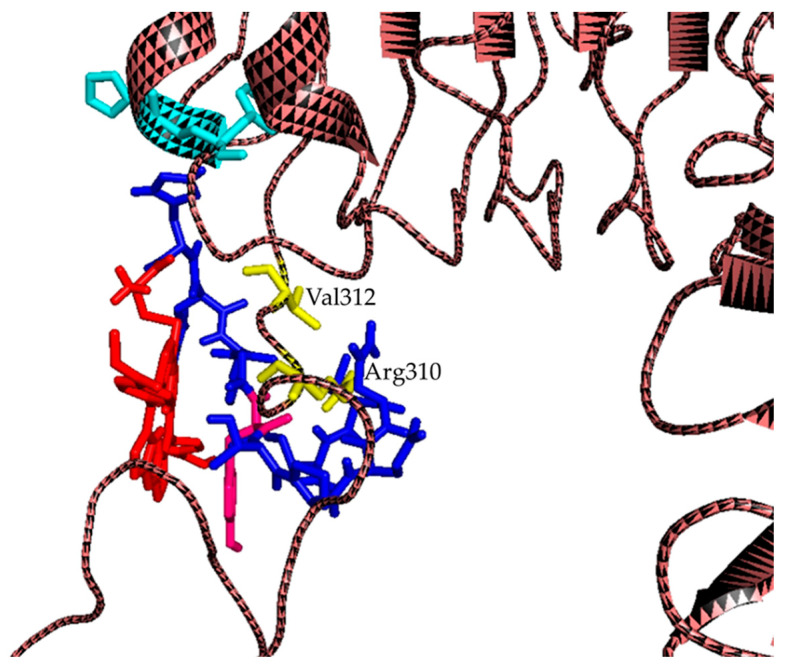
Proposed model of interaction of EGFR with conjugate **7**. In yellow, the aa **Arg310** and **Val312** of the EGFR, while in pink **Tyr6** of the peptide. The porphyrin moiety is colored in red. The EGFR protein is shown in a cartoon representation.

Microwave-assisted Fmoc-SPPS was employed to synthesize peptides **3**–**5**. Conjugation via thiol–nitro aromatic nucleophilic substitution was performed under conditions previously optimized in our group for the displacement of an aromatic fluoride by a thiol [48]. Preliminary experiments using N-acetylcysteine as the model nucleophile demonstrated that the displacement reaction commenced within minutes in DMSO, in the presence of cesium carbonate (Cs_2_CO_3_), as the base and proceeded smoothly to completion in 1 h. Encouraged by these results, we extended the thiol-based nucleophilic aromatic substitution strategy to conjugate peptides **3**–**5** to porphyrin **2** (Figure 1B). The conjugation reactions were carried out using 1 equivalent of peptide and 2 equivalents of base, with reaction progress monitored by LC–MS. The reactions proceeded to afford the desired peptide–porphyrin conjugates were obtained in good isolated yields (~80%) within 24 h. Increasing the reaction time beyond 30 h resulted in the formation of multiple by-products, which LC–MS analysis suggested arising from peptide backbone degradation (Figure S2). Following precipitation of unreacted porphyrin with cold diethyl ether, the target conjugates were isolated by evaporation of the supernatants. The structures of the resulting conjugates are shown in Figure 1 and Figure S3. Molecular docking studies performed on conjugates **6**–**8** demonstrated their ability to bind to EGFR, preserving the key interaction observed with the free peptide, namely a hydrogen bond between **Tyr6** of the peptide moiety and the side chain of **Arg310** on the receptor (Figure 2).

It is important to note that the docking analysis of the conjugates is inherently limited by the presence of the porphyrin moiety, which cannot be reliably accommodated within the docking simulation due to its size and conformational complexity. However, based on previously reported studies with other types of PS, it can be hypothesized that the porphyrin may occupy a spatial region overlapping or adjacent to the peptide binding pocket, potentially contributing to or stabilizing the interaction with EGFR [37].

#### 2.1.3. Photostability

**Figure 3:**
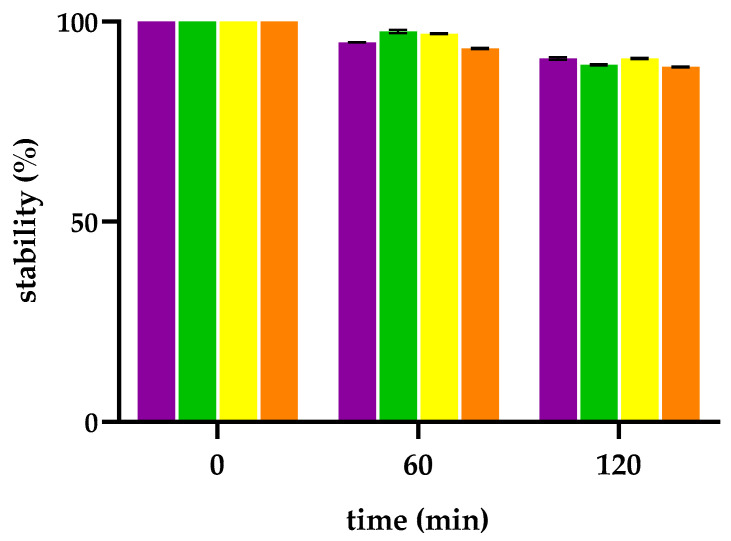
Residual photostability of porphyrin **2** (purple) and its peptide conjugates, **6** (green), **7** (yellow), **8** (orange), after irradiation with a 500 W tungsten halogen lamp up to 120 min.

Compounds incorporating photosensitizing chromophores, such as tetrapyrrolic macrocycles, are susceptible to ROS-mediated photodegradation upon irradiation. Photostability of PSs is a critical parameter for achieving safe, effective, and precise PDT. It ensures sustained generation of ROS, minimizes the formation of potentially toxic or mutagenic degradation products, and preserves the structural and photophysical integrity of the PS during systemic circulation and tumor accumulation. High photostability is particularly important in delayed-activation protocols, enabling selective localization in target tissues. Moreover, it supports consistent dosing and reduces off-target phototoxicity caused by premature activation under ambient light [57,58]. Thus, before assessing the photodynamic activity of the conjugates in TNBC cell lines, the photostability of **2** and conjugates **6**, **7**, and **8** was evaluated under the same conditions employed for the cell viability assays and the other biological studies reported in the present study. This was carried out by monitoring the decrease in the intensity of the Soret band as well as time-dependent changes in the absorption spectra, which could indicate structural alterations or degradation of the porphyrin macrocycle. The results obtained show that the Soret bands of porphyrin **2** and the three conjugates retain approximately 90% intensity following 2 h exposure to a 500 W tungsten-halogen lamp, indicating a good stability of the four compounds to irradiation (Figure 3).

To assess whether the peptide moiety underwent photoinduced degradation, samples of porphyrin **2** and its conjugates **6**–**8** were recovered and analyzed by HPLC at the end of the irradiation period. No shifts in retention times were observed, nor did any additional peaks appear in the chromatograms, confirming the structural integrity of both the porphyrin core and the peptide chains under the irradiation conditions employed.

### 2.2. Biological Results

#### 2.2.1. Cell Lines and EGFR Expression

The MDA-MB-453 and MDA-MB-231 breast cancer cell lines were selected to evaluate the anticancer efficacy of the porphyrin–peptide conjugates, as they are representative models of TNBC. Notably, MDA-MB231 cells exhibit elevated EGFR expression compared to MDA-MB453 cells, providing a suitable model for evaluating EGFR-targeted photodynamic activity [59,60]. This differential EGFR expression was further confirmed in the present study by Western blot analysis (Figure S4).

#### 2.2.2. Cellular Uptake

Previous studies have reported a correlation between photodynamic efficacy and the intracellular accumulation of photosensitizers [61]. To investigate this relationship, the intrinsic fluorescence of porphyrin **2** was exploited to monitor compound uptake. TNBC cell lines were incubated with an equimolar concentration (100 nM) of porphyrin **2** and conjugates **6**, **7**, and **8** for 24 h under dark conditions at either 37 °C or 4 °C. Incubation at 4 °C was employed to evaluate uptake via passive transport mechanisms, such as diffusion, facilitated diffusion, filtration, and osmosis, as active transport processes are largely inhibited at this temperature [62]. Cellular uptake values obtained were expressed as mean fluorescence intensity (MFI).

**Figure 4:**
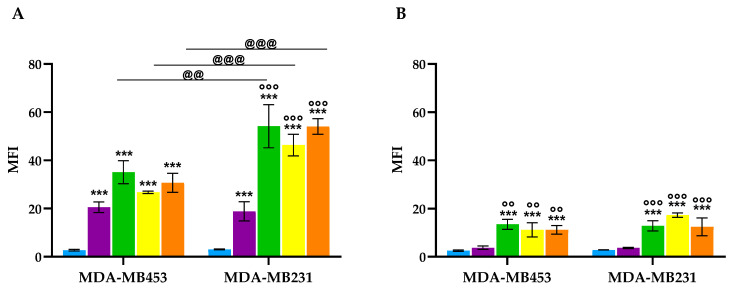
Cellular uptake following treatment of MDA-MB453 and MDA-MB231 cells with 100 nM of **2** (purple), **6** (green), **7** (yellow), and **8** (orange) and flow cytometric analysis. Experiments were performed at 37 °C (**A**) and 4 °C (**B**). Mean ± SD of 3–4 independent experiments. *** *p* < 0.001 vs. Ctrl (light blue); °° *p* < 0.01, °°° *p* < 0.001 vs. **2**; ^@@^ *p* < 0.01, ^@@@^ *p* < 0.001.

As shown in Figure 4, at 37 °C, all conjugates exhibited significantly higher cellular uptake in the EGFR-overexpressing MDA-MB231 cells compared to the non-overexpressing MDA-MB453 cells. Moreover, in the former cell line the accumulation of conjugates **6**–**8** was significantly greater than that of porphyrin **2**, whereas in MDA-MB453 cells no significant differences in uptake were observed among the conjugates and porphyrin **2**. When the experiment was conducted at 4 °C, a marked reduction in cellular uptake was observed in both cell lines, and no significant differences in uptake were detected between them, indicating that the internalization process is temperature-dependent. Despite the overall decrease in compound accumulation at this temperature, the uptake of conjugates **6**–**8** remained significantly higher than that of porphyrin **2** in both cell lines.

Overall, these findings support the hypothesis that conjugates **6**–**8** are internalized via active transport mechanisms and preferentially target EGFR-overexpressing TNBC cells.

In addition to the extent of PSs uptake by cells, their subcellular localization is a critical factor that determines the type and degree of cell death triggered and influences the overall PDT effectiveness [18,63,64]. ROS produced upon photoactivation have a limited diffusion range; therefore, the site of ROS generation largely determines the primary targets of damage and the downstream signaling pathways leading to cell death. For example, PSs localized in mitochondria often induce apoptosis via mitochondrial membrane permeabilization and cytochrome c release, while those accumulating in lysosomes can cause lysosomal membrane permeabilization, leading to either apoptosis or necrosis, depending on the extent of damage. Endoplasmic reticulum (ER)-localized PSs may activate ER stress-mediated apoptotic pathways or promote immunogenic cell death. Additionally, nuclear localization can lead to direct DNA damage, though this is less common due to the generally poor nuclear penetration of most PSs [63,64]. Although subcellular localization was not the primary focus of this study, a preliminary experiment was conducted to assess the intracellular distribution of one of the conjugates (i.e., conjugate **7**) using MitoView^®^ and LysoView^®^ as organelle-specific markers. Confocal microscopy analysis of MDA-MB-453 and MDA-MB-231 cells revealed a diffuse fluorescence signal distributed throughout both the cytoplasm and nucleus, suggesting a broad intracellular localization of the conjugate. Despite this widespread distribution, mitochondrial accumulation of conjugate **7** was observed in both cell lines. Notably, MDA-MB-453 cells exhibited a more extensive and structurally organized mitochondrial network relative to MDA-MB-231 cells, resulting in a higher degree of co-localization with the mitochondrial marker in the former (Figure S5). The broad and non-specific intracellular distribution of the photosensitizer, however, precluded a conclusive evaluation of its co-localization with lysosomes.

#### 2.2.3. Photodynamic Activity

The effect of porphyrin **2** and its conjugates (**6**–**8**) on the viability of TNBC cell lines was evaluated using the MTT assay. Some researchers have noted that MTT results alone may not be sufficient to thoroughly assess photokilling effectiveness, and that clonogenic assays should also be conducted for this purpose The MTT assay is known to assess cellular metabolic activity, which, although not a direct measurement of viability, is widely accepted as an indirect indicator of cell viability in the scientific community. Notably, recent studies have highlighted certain limitations of the assay in specific contexts where MTT readings may not exhibit a linear correlation with cell number. However, these findings emphasize the importance of cautious data interpretation rather than justifying a wholesale rejection of the assay’s applicability [65,66]. Thus, numerous peer-reviewed studies continue to regard the MTT assay a valid and valuable tool for detecting treatment-induced changes in cell viability, and it remains widely employed for this purpose, including in the context of in vitro PDT research [67,68].

Both the light-dependent effects and the intrinsic (dark) cytotoxicity of the compounds were investigated. For assessment of dark toxicity, cells were treated with a 10-fold higher concentration of each compound in the absence of irradiation. All compounds exhibited negligible dark toxicity (Figure S6), indicating the need for photoactivation to elicit a cytotoxic effect.

**Figure 5:**
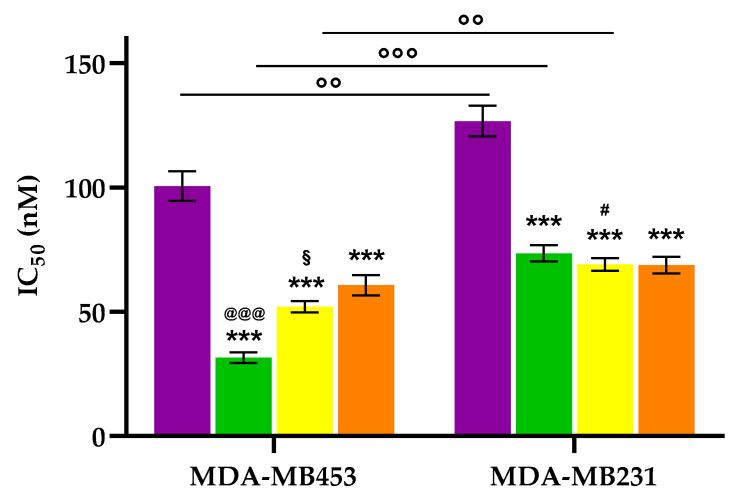
IC_50_ values obtained in MDA-MB453, and MDA-MB231 cell lines following 24 h treatment with porphyrin **2** (purple), **6** (green), **7** (yellow), and **8** (orange), 2 h photoactivation, 24 h incubation in drug-free medium, and MTT assay (mean ± SD of 5 independent experiments. *** *p* < 0.001 vs. **2**; ^@@@^ *p* < 0.001 vs. **7** and **8**; # *p* < 0.05 vs. **6**; ^§^ *p* < 0.05 vs. **8**; °° *p* < 0.01, °°° *p* < 0.001).

Half-maximal inhibitory concentration (IC_50_) values for the conjugates were determined from dose–response curves and compared to those of the reference compound **2** (Figure 5).

Following photoactivation, all compounds showed photodynamic activity across all tested cell lines. All conjugates demonstrated significantly higher photodynamic activity compared to porphyrin **2** in the TNBC cell lines. Among the TNBC models, the conjugates had similar effectiveness in MDA-MB231 cells; however, in MDA-MB453 cells, conjugate **6** was significantly more potent than conjugates **7** and **8**. Interestingly, despite MDA-MB453 cells expressing lower levels of EGFR than MDA-MB231 cells, they showed greater sensitivity to all tested compounds. This pattern mirrors what is often seen in clinical responses to TNBC therapies. Although many TNBCs overexpress EGFR, a large portion do not respond to EGFR-targeted treatments [57,58]. A likely explanation is that most TNBCs are not solely dependent on EGFR signaling for survival, emphasizing the molecular diversity of the disease and the need for multi-faceted therapeutic approaches [58,59]. Overall, the link between EGFR levels and response to different EGFR inhibitors remains under discussion. Some studies indicate that TNBC cells with low EGFR protein levels, including MDA-MB453 cells, are more resistant to EGFR inhibitors compared to those with higher levels [58,60], while others suggest the opposite trend, with greater sensitivity in cells with lower EGFR levels [61].

Additionally, the data indicate that the direct attachment of the tetrapyrrole core to the peptide chain (i.e., without a meso-aryl spacer) does not hinder receptor binding or photodynamic activity, thereby validating the predictions made by the molecular docking studies.

#### 2.2.4. ROS Production

As previously mentioned, ROS generation by the PS is the main mechanism underlying PDT-induced localized cytotoxicity and tissue damage. Upon activation by light, the excited-state PS can engage in two primary pathways. In Type I photochemical reactions, the excited PS undergoes electron or proton transfer with biological substrates such as unsaturated lipids, proteins, or nucleic acids, forming unstable radical species. In the presence of molecular oxygen, this results in the production of ROS, including superoxide anion (O_2_•^−^), hydroxyl radical (•OH), and hydrogen peroxide (H_2_O_2_). Alternatively, in Type II reactions, the excited PS transfers energy directly to ground-state molecular oxygen, generating singlet oxygen (^1^O_2_). Therefore, the efficacy of photodynamic therapy is closely correlated with the extent of ROS generation induced by the photosensitizer [69].

**Figure 6:**
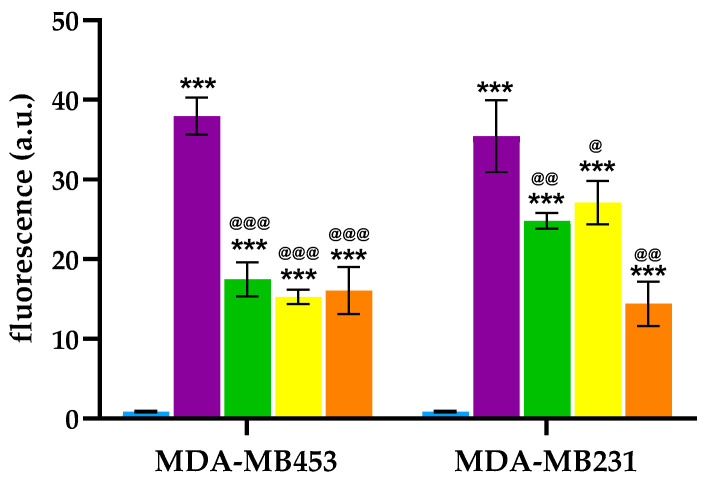
ROS levels in MDA-MB453 and MDA-MB231 cells after PDT with **2** (purple), **6** (green), **7** (yellow), and **8** (orange). In control samples (Ctrl: light blue), PS treatment was omitted (Mean ± SD of 3 independent experiments. *** *p* < 0.001 vs. Ctrl; ^@@@^ *p* < 0.001, ^@@^ *p* < 0.01, ^@^ *p* < 0.05 vs. **2**).

In the present study, intracellular ROS generation was measured using fluorescence microscopy after 24 h of treatment with **2**, **6**, **7**, and **8** at their respective IC_50_ concentrations, followed by a 2 h irradiation period in the presence of the fluorescent probe 2′,7′-dichlorodihydrofluorescein diacetate (DCFH-DA), which detects general oxidative stress within cells. Porphyrin **2** produced higher levels of intracellular ROS in MDA-MB453 and MDA-MB231 cells compared to its peptide-conjugated derivatives (Figure 6). However, no significant differences were observed among the three peptide–porphyrin conjugates in their ability to induce ROS production in both TNBC cell lines.

The apparent paradox of reduced ROS production yet increased PDT efficacy for the conjugates can be explained by considering that, while overall ROS measurement provides useful information about the photophysical performance of a photosensitizer, the biological outcome of PDT is not determined solely by the total amount of ROS produced but also by their location, type, and duration [70]. Conjugation to nanoparticles or peptides promotes selective accumulation and retention at vulnerable subcellular sites (e.g., mitochondria, lysosomes, or the plasma membrane) and increases local photosensitizer concentration [71,72]. Therefore, even with extremely short ROS lifetimes and diffusion distances, a lower total ROS output can cause disproportionately greater local damage when generated near critical organelles or membranes [73]. Conjugation can also shield the PS from extracellular or intracellular quenchers, change ROS types toward more cytotoxic forms, and enable activatable behaviors (e.g., pH- or enzyme-triggered disassembly) that protect off-target tissues while inducing potent local phototoxicity [74]. The enhanced cellular uptake of the conjugates could also contribute to the increased PDT effectiveness observed. These combined effects have been consistently reported for peptide- and nanoparticle-based PS delivery systems and explain how efficacy can rise even when bulk ROS tests show a lower overall signal [75]. In addition, it should be noted that MTT assay directly evaluates mitochondrial activity, which can remain temporarily functional in cells destined to die, and may thus contribute to the apparent discrepancy between ROS production and MTT results.

#### 2.2.5. Cell Death Induction

The response of cancer cells to PDT is influenced by multiple factors, including the histological origin of the tumor, the photophysical and photochemical characteristics of the PS, its biodistribution within tissues, cellular uptake, subcellular localization, and the parameters of light exposure, particularly irradiation dose [76,77,78]. Importantly, photodynamic reactions can concurrently activate multiple cell death pathways, which play a critical role in determining the overall therapeutic efficacy of PDT [77,79]. Under optimized PDT protocols, regulated cell death mechanisms are observed. Among these, apoptosis and autophagy have been the most extensively characterized and are recognized for their dual roles in promoting both cytotoxic and pro-survival responses [21]. The molecular underpinnings of PDT-induced apoptosis and autophagy have been comprehensively reviewed in the literature [21,77,80,81]. In contrast, when PDT is administered under aggressive conditions, such as high PS concentrations or high-intensity irradiation, cancer cells often undergo rapid, unregulated necrotic cell death, also known as accidental necrosis [77,79].

**Figure 7:**
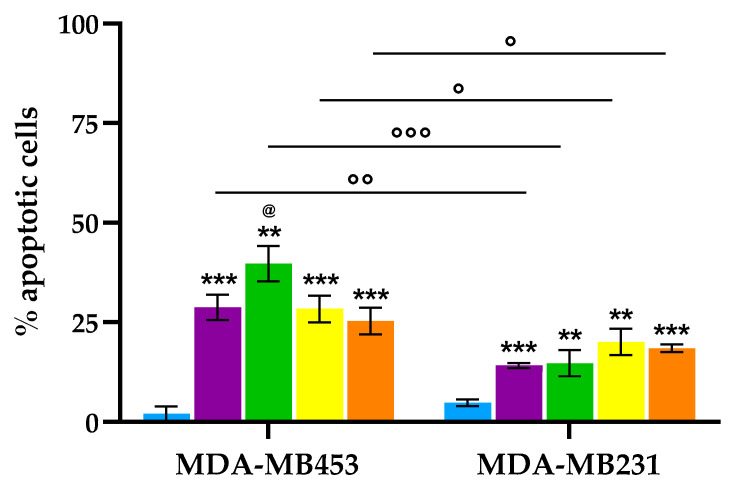
Percentage of apoptotic cells after PDT (Ctrl: light blue; **2**: purple; **6**: green; **7**: yellow; **8**: orange; mean ± SD of **3**–**5** independent experiments. *** *p* < 0.001, ** *p* < 0.01 vs. Ctrl; ^@^ *p* < 0.05 vs. all the other PSs; °°° *p* < 0.001, °° *p* < 0.01, ° *p* < 0.05).

Our findings demonstrate that treatment with equitoxic concentrations of the tested porphyrins, corresponding to their respective IC_50_ values, followed by photoactivation, led to a significant increase in the proportion of apoptotic MDA-MB453 and MDA-MB231 cells compared to untreated controls (Figure 7). Notably, a differential apoptotic response was observed between the two cell lines. Consistent with the MTT assay results, MDA-MB453 cells exhibited a higher percentage of apoptosis, with conjugate **6** emerging as the most potent inducer of apoptotic cell death. In contrast, MDA-MB231 cells displayed comparable levels of apoptosis across all treatments, suggesting a lower sensitivity or differential mechanism of response to the porphyrin-based PDT in this cell line.

**Figure 8:**
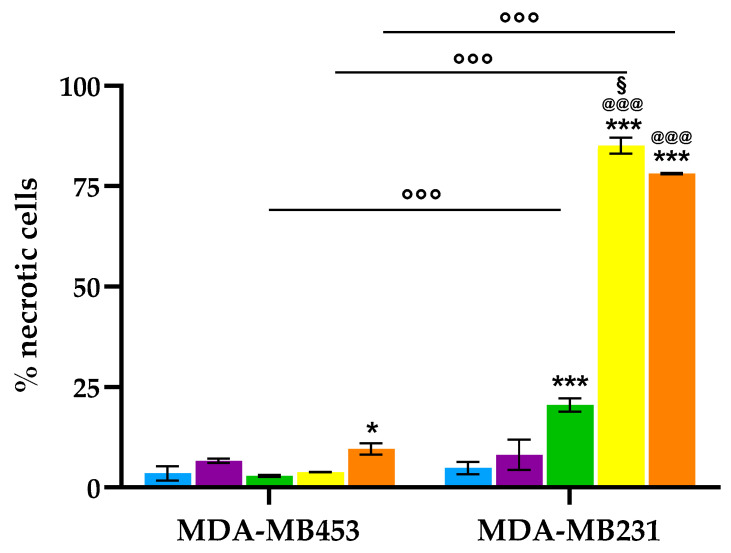
Percentage of necrotic cells after PDT (Ctrl: light blue; **2**: purple; **6**: green; **7**: yellow; **8**: orange; mean ± SD of **3**–**5** independent experiments. *** *p* < 0.001, * *p* < 0.05 vs. Ctrl; ^@@@^ *p* < 0.001 vs. **2** and **6**; ^§^ *p* < 0.05 vs. **8**; °°° *p* < 0.001).

As shown in Figure 8, a significant increase in necrotic cell death, relative to control cells, was observed exclusively in the MDA-MB231 cell line treated with the three conjugates, with **7** and **8** being the most effective in inducing necrosis, with necrotic cell populations reaching up to 80%. In contrast, the MDA-MB453 cell line exhibited minimal necrotic response, with only compound **8** inducing a statistically significant increase in necrosis; however, the percentage of necrotic cells in this case did not exceed 10%.

Overall, the apoptosis and necrosis results show that MDA-MB231 cells primarily undergo necrotic cell death after PDT with the tested compounds, while MDA-MB453 cells display a greater tendency to activate apoptotic pathways. This difference in susceptibility suggests a cell line-specific response to photodynamic treatment and highlights the importance of tumor subtype in determining the dominant mode of cell death.

The cellular response to photodynamic treatment depends on several factors. Among these, the subcellular location of the photosensitizer is especially crucial because it determines the primary site of photodamage and influences the subsequent cell death pathways [63,82]. It is well known that organelles like mitochondria, endoplasmic reticulum, lysosomes, and the plasma membrane are involved differently in cell death signaling. The site where the photosensitizer accumulates affects whether apoptotic or necrotic pathways are favored. Photosensitizers in mitochondria tend to trigger rapid apoptosis, while those in endoplasmic reticulum and lysosomes can induce either immunogenic cell death, apoptosis, or necrosis [82]. Therefore, the different responses seen in the two TNBC cell lines in the present study may partly result from variations in the subcellular localization of the photosensitizer. Specifically, the higher mitochondrial co-localization in MDA-MB453 cells could explain their greater tendency for apoptotic cell death after PDT.

Induction of autophagy occurrence was evaluated by assessing the expression levels of the well-established autophagic marker LC3-II through Western blot analysis. The LC3 protein yields two distinct immunoreactive bands: LC3-I (~16 kDa), representing the cytosolic, non-lipidated form, and LC3-II (~14 kDa), the lipidated form generated by conjugation of LC3-I with phosphatidylethanolamine. LC3-II is specifically associated with autophagosomal and isolation membranes, and its accumulation serves as a hallmark of autophagosome formation and autophagic activity [83].

**Figure 9:**
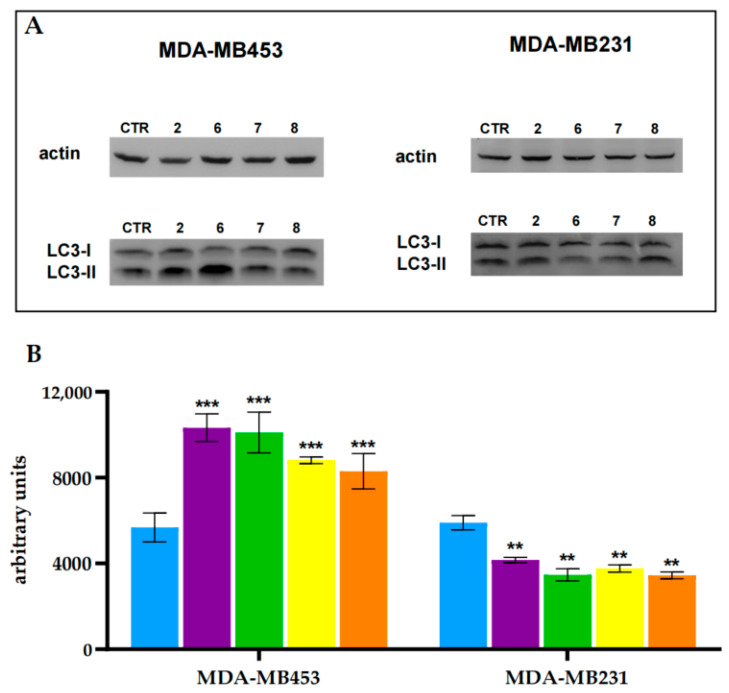
LC3-II protein levels in MDA-MB453 and MDA-MB231 cell lines treated with equitoxic concentrations of **2** (purple), **6** (green), **7** (yellow), and **8** (orange), corresponding to their respective IC_50_, 2 h irradiation, and 24 h incubation in drug-free medium. Representative immunoblot images (**A**), and corresponding densitometric analysis (**B**). Densitometric quantification is based on two independent experiments. Statistical significance is indicated as *** *p* < 0.001, ** *p* < 0.01 compared to untreated control (Ctrl, light blue). Uncropped blot images are provided in Figure S6.

MDA-MB453 cells, all treatments resulted in a significant upregulation of autophagy, as evidenced by increased LC3-II protein levels, irrespective of the compound employed. In contrast, MDA-MB231 cells exhibited no significant increase in LC3-II expression following treatment, indicating a limited or absent autophagic response under the same conditions (Figure 9).

In general, PDT elicits its therapeutic effects predominantly through the induction of apoptosis and necrosis. Autophagy, however, represents a context-dependent response that may function as either a cytoprotective mechanism, facilitating cellular adaptation to photodynamic stress, or as a pathway contributing to cell death. The direction of this autophagic response is influenced by multiple factors, including the chemical nature and subcellular localization of the photosensitizer, as well as the intrinsic characteristics of the target cell type [84]. Distinguishing between these roles requires dedicated experiments, such as pharmacological modulation of autophagy or genetic silencing of key autophagy-related genes, which were beyond the scope of this work.

#### 2.2.6. Antimigratory Effects

Tumor cell motility and invasiveness are key drivers of metastasis and constitute major obstacles to effective cancer therapy. These behaviors are frequently mediated by dysregulated responses to extracellular stimuli, including growth factors and cytokines. Among these signaling molecules, EGF has been shown to enhance tumor cell motility and invasiveness in several cancer models by activating its receptor, EGFR [85,86,87]. Furthermore, TNBC exhibits high migratory capacity, contributing to its elevated metastatic potential and further restricting effective therapeutic options [88].

Recent findings suggest that a significant number of PSs, including porphyrin-like ones, possess the ability to inhibit cellular migratory activity [89,90,91], making them a promising therapeutic option for highly aggressive tumors, including TNBC.

The scratch wound healing assay was performed to evaluate the potential antimigratory effects of porphyrin **2** and its derivatives (compounds **6**–**8**). Wound closure was monitored over time using a microscope equipped with a digital imaging system. Representative images were captured at defined time points to assess cell migration. For each condition, the percentage of remaining open wound area was quantified to determine the extent of cell migration.

**Figure 10:**
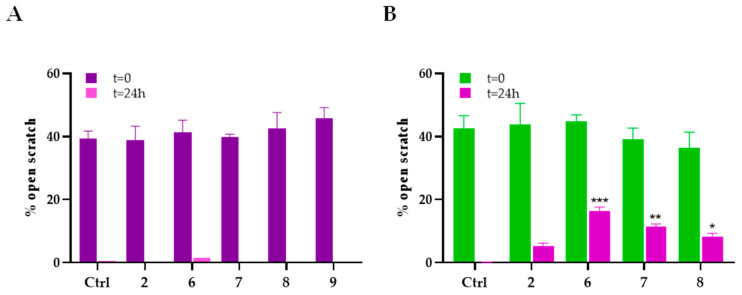
Migratory activity of MDA-MB453 (**A**) and MDA-MB231 (**B**) cells following treatment with **2**, **6**–**8** for 24 h, scratch formation, 2 h irradiation, and incubation for 24 h in drug-free medium at 37 °C. Pictures of the scratch wound were taken immediately following the irradiation step (t_0_) and after 24 h, through a camera connected to an Olympus IX81 microscope (mean ± S.D. of 3 independent experiments; * *p* < 0.05, ** *p* < 0.01, *** *p* < 0.001 vs. **2**).

Figure 10 show the percentage of open wound areas in MDA-MB453 and MDA-MB231 cells obtained 24 h after PDT of the cells incubated for 24 h with subtoxic concentrations of the tested compounds (Figure S7). Distinct responses were observed between the two cell lines. In MDA-MB453 cells, which exhibit low EGFR expression, none of the tested compounds, either targeted or non-targeted, significantly inhibited cell migration following PDT. This suggests that in the absence of substantial EGFR expression, the efficacy of the photosensitizers in disrupting cellular motility is limited. The lack of response may correlate with the reduced cellular uptake observed. In contrast, MDA-MB231 cells, characterized by high levels of EGFR expression, exhibited a significant reduction in migration following PDT with all tested compounds. Notably, the derivatives functionalized with EGFR-targeting peptides demonstrated a significantly greater inhibitory effect on cell migration compared to the non-targeted porphyrin **2**. This highlights the importance of targeted delivery in enhancing the specificity and therapeutic impact of the treatment, likely due to increased accumulation and retention of the photosensitizers in EGFR-rich cells. In the absence of the photoactivation step, none of the compounds exhibited inhibitory effects on cell migration. This confirms that the observed effects are PDT-dependent and not due to off-target effects of the compounds themselves at the concentrations used. It reinforces the specificity and safety profile of the treatment approach, where the therapeutic effect is tightly controlled by light activation.

## 3. Materials and Methods

### 3.1. General Remarks

^1^H NMR spectra were recorded either on a Bruker AVA400 spectrometer (600 MHz) (Bruker Corporation, Coventry, UK). ^13^C NMR spectra were recorded on a Bruker AVA400 spectrometer operating at 151 MHz. CDCl_3_ was used as the solvent. Chemical shifts are expressed in parts per million (ppm) with respect to CDCl_3_ (7.26) and are reported as s (single), d (doublet), t (triplet), and m (multiple). Coupling constants were expressed in Hertz. Data analyses were performed using TopSpin 3.1, Bruker UK Ltd. (Coventry, UK). LC-MS was performed on an Agilent 1260 Infinity II LC with Agilent 6530 Accurate-Mass QToF spectrometer, equipped with Agilent ZORBAX SB-C18 Stable Bond Analytical (particle size 5 μm, 4.6 × 150 mm) from Agilent Technologies UK Limited (Agilent Technologies LDA UK Limited, Lakeside, UK) with a binary eluent system comprising MeCN/H_2_O (30 min gradient: 90–10% with 0.1% di FA). Mass spectrometry was conducted in positive ion mode (*m*/*z* range: 50–3200) using a fragmenter voltage of 150 V, a gas temperature of 325 °C (flow 10 L/min), and a gas temperature of the sheath of 400 °C (flow 11 L/min).

The UV-Vis absorption spectrum was measured using a Perkin-Elmer Lambda 10 spectrophotometer (PerkinElmer Inc., High Wycombe, UK).

Thin-layer chromatography (TLC) was performed using pre-coated sheets of 60 F254 silica gel (thickness 0.2 mm), while silica gel 60 (70–230 mesh, Merck, Milan, Italy) was used for the chromatographic column separations.

Methyl 4-formylbenzoate, p-Chloranil, Cs_2_CO_3_, I_2_, and AgNO_2_ are Merck commercial products used as received. All the solvents used in the synthesis, as well as those used for elution in the chromatographic column and for the analyses, are Merck products.

Pyrrole and DCM used to synthesize the porphyrin were freshly distilled directly in the reaction flask.

All Fmoc-aa with standard side chain protecting groups, Rink Amide Pro-Tide resin, and Oxyma Pure were purchased from CEM UK Ltd. (CEM Corporation, Buckingham, UK).

For compounds **2** and conjugates **6**–**8**, 1 mM stock solutions in DMSO were prepared, once the necessary analyses were carried out to confirm their composition.

For the chemical and biological analyses a 500 W tungsten white halogen lamp was used. The tungsten lamp irradiation device was positioned above the target area at a distance that ensures even exposure. For this type of lamp, a cooling system is necessary to prevent overheating, so a flow water filter was placed between the lamp and the irradiated area. The lamp has an irradiance of 22 mW/cm^2^ (an average value determined between 380 and 780 nm with a Licor-1800 spectroradiometer, Li-COR, Bad Homburg, Germany), equating to 158 J/cm^2^ of fluence over 2 h.

### 3.2. Synthesis

#### 3.2.1. The Synthesis of 5,15-Bis(4-carbomethoxyphenyl)porphyrin (**1**)

To 500 mL of freshly distilled DCM were added 5.80 mmol of dipyrromethane (850 mg) obtained as reported in the literature [46], 3.00 mmol of methyl 4-formylbenzoate (490 mg) and 24 drops of TFA. The reaction was kept at room temperature (RT) overnight. After TLC control (SiO_2_; DCM:MeOH = 95:5) to verify the disappearance of methyl 4-formylbenzoate, 4.37 mmol of *p*-chloranil (1.07 g) was added, and the reaction was refluxed for 3 h to obtain the porphyrin of interest following the complete oxidation of the porphyrinogen. At the end of the 3 h of reflux, the reaction was left at RT ON. After removing the solvent, the crude product was purified by means of a chromatographic column (SiO_2_; DCM:MeOH = 95:5). The product obtained was precipitated in DCM over MeOH and filtered to recover a solid crystalline product of purple color (1.07 g, 1.85 mmol, 61.66% yield).

Chemical Formula: C_36_H_26_N_4_O_4_. Molecular Weight: 578.63 g/mol. MS (ESI): M^+^ found: 579.20. Purity 98%. ^1^H NMR (600 MHz, CDCl_3_): δ −3.11 (2H, s), 4.14 (6H, s), 8.37 (4H, d, J = 4.8), 8.50 (4H, d, J = 4.8), 9.04 (4H, d, J = 7.8), 9.43 (4H, d, J = 7.8), 10.36 (2H, s).

#### 3.2.2. The Synthesis of 10-Nitro-5,15-bis(4-carbomethoxyphenyl)porphyrin (**2**)

In a 250 mL reaction flask, 0.20 mmol of **1** (116 mg) was dissolved in 100 mL of dry DCM and 40 mL of dry MeCN. Once a homogeneous solution was obtained, a solution containing 0.2 mmol of I_2_ (51 mg) in dry DCM was added and stirred at RT for 10 min. A 0.36 mmol solution of AgNO_2_ (55 mg) dissolved in dry MeCN was then added. Immediately, a yellow/gray precipitate was formed. The reaction was left under stirring for 40 min. The reaction was then centrifuged at 5000 rpm for 10 min at the end of which the supernatant was recovered and dried. A violet-colored solid was obtained (106 mg, 0.17 mmol, 85% yield).

Chemical Formula: C_36_H_25_N_5_O_6_. Molecular Weight: 623.63 g/mol. MS (ESI): M^+^ found: 624.18. HPLC retention time 14′41″; purity 97%. ^1^H NMR (600 MHz, CDCl_3_): δ −3.01 (2H, s), 4.14 (6H, s), 8.30 (4H, d, J = 7.6), 8.50 (2H, d, J = 7.2), 8.44–9.00 (4H, m), 9.35–9.38 (4H, m), 10.36 (1H, m).^13^C NMR (151 MHz, CDCl_3_): δ 55.6, 120.80, 128.3, 128.5, 129.5, 130.1, 131.4, 132.7, 134.5, 145.3, 167.1.

#### 3.2.3. Peptides (**3**–**5**)

Peptide sequences **3** (NCVVGYIGERCQ), **4** (CHWYGYTPENVI) and **5** (CHWYGYTPQNVI) were obtained using a CEM Liberty Blue microwave-assisted peptide synthesizer. The synthesis was carried out on Rink amide resin Pro-Tide (180 mg, 0.56 mmol/g loading; 0.1 mmol), using Fmoc-aa (0.2 M in DMF) as the building blocks, DIC (1 M stock solution in DMF) as activator, Oxyma Pure^®^ (1 M stock solution) as racemization suppressor and piperidine (20% *v*/*v* in DMF) as deprotection agent. The coupling procedure generally involved single coupling (2 min at 90 °C) followed by Fmoc-deprotection (2 min at 90 °C). Cysteine was coupled under milder conditions (10 min at 50 °C) while arginine was coupled using a modified double coupling cycle. Upon synthesis completion, the resin was transferred into 10 mL syringes equipped with frits and washed with Et_2_O. Cleavage and deprotection were carried out by treatment with TFA, TIPS, and H_2_O (8:1:1 *v*/*v*) with shaking at RT for 4 h. The solution was evaporated to a small volume, and the peptides were precipitated by dropwise addition to cold Et_2_O followed by centrifugation (13,500 rpm, 5 min). The precipitate was resuspended in Et_2_O and centrifuged again. The resulting white solid was analyzed by LC-MS to identify purity and confirm identity. For the LC-MS analysis, the peptide was dissolved in MeOH with 0.1% FA *v*/*v* and analyzed as reported.

*Peptide ***3*** (NCVVGYIGERCQ)*. Chemical Formula: C_55_H_89_N_17_O_18_S_2_. Molecular Weight: 1341.61 g/mol. MS (ESI): M^+^ found: 1342.60. HPLC retention time 7′53″; purity 99%.

*Peptide ***4*** (CHWYGYTPENVI)*. Chemical Formula: C_69_H_92_N_16_O_19_S. Molecular Weight: 1481.65 g/mol. MS (ESI): M^+^ found: 1482.65. HPLC retention time 8′31″; purity 99%.

*Peptide ***5*** (CHWYGYTPQNVI)*. Chemical Formula: C_69_H_93_N_17_O_18_S. Molecular Weight: 1480.66 g/mol. MS (ESI): M^+^ found: 1481.66. HPLC retention time 8′27″; purity 98%.

#### 3.2.4. Porphyrin–Peptide Conjugates (**6**–**8**)

First, 0.05 mmol of **2** (31 mg), 0.05 mmol of peptide, and 0.10 mmol of Cs_2_CO_3_ (32 mg) were dissolved in 1.5 mL of DMSO and subjected to magnetic stirring at RT for 24 h, as reported in the literature [47]. At predetermined times, samples of 20 μL were carried out to be analyzed in LC-MS after dilution in 1 mL of MeCN + 0.1% FA. At the end of 24 h, 5.0 mL of MeCN + 1% of FA were added to the reaction. The reaction was then precipitated in cold Et_2_O and centrifuged at 5000 rpm for 15 min at 4 °C. The supernatant was dried and analyzed by LC-MS.

*Conjugate*** 6*** (***2** + *peptide* **3***)*. (80.12% yield). Chemical Formula: C_91_H_113_N_21_O_22_S_2_. Molecular Weight: 1917.15 g/mol. MS (ESI): M^+^ found: 1918.13. HPLC retention time 11′33″; purity 98%.

*Conjugate*** 7*** (***2** + *peptide* **4***)*. (80.00% yield). Chemical Formula: C_105_H_116_N_20_O_23_S. Molecular Weight: 2058.26 g/mol. MS (ESI): M^+^ found: 2059.23. HPLC retention time 12′38″; purity 97%.

*Conjugate*** 8*** (***2** + *peptide* **5***)*. (80.42% yield). Chemical Formula: C_105_H_117_N_21_O_22_S. Molecular Weight: 2057.28 g/mol. MS (ESI): M^+^ found: 2058.25. HPLC retention time 12′35″; purity 98%.

### 3.3. Modeling and Docking

The 3D structures of peptides **3**–**5** and conjugates **6**–**8** were obtained using the Avogadro 1.2 molecular modeling software and subsequently used for docking studies.

PDB entry 1nql7 was used for the 3D structure of EGFR, which represents the extracellular domain of EGFR in an inactive (low pH) complex with EGF. For analysis, water and other molecules were removed from the file.

A grid containing aa Arg310 and Val312 was created for the docking of the peptides, as reported in previous studies published in the literature [37,91]. For conjugates the grid was created to contain aa His334 and Phe335 in addition to aa Arg310 and Val312.

Docking was performed using Autodock Vina 1.1.2. The lowest energy docked structure is represented using PyMol 3.1 software (Schrodinger LLC, Portland, OR, USA).

### 3.4. Photobleaching

A 10 μM solution of each porphyrin–peptide conjugate was prepared in PBS. The resulting solutions were irradiated for 2 h using a tungsten–halogen lamp. At predetermined time intervals, aliquots were collected and analyzed by UV–Vis spectrophotometry. The extent of photodegradation was quantified as the percentage decrease in absorbance relative to the initial absorbance at time zero (t_0_).

### 3.5. Biological Studies

#### 3.5.1. Cell Lines and Experimental Conditions

TNBC tumor lines MDA-MB453 and MDA-MB231 were obtained from the American Type Culture Collection (Rockville, MD, USA) and incubated under standard culture conditions at 37 °C in a humidified atmosphere with 5% CO_2_. The MDA-MB453 cell line was grown in Dulbecco’s Modified Eagle Medium (DMEM, Euroclone, Milan, Italy), and MDA-MB231 cells were maintained in Roswell Park Memorial Institute (RPMI) 1640 (Euroclone) medium. Cell culture media were supplemented with 10% fetal bovine serum, 1% glutamine, 0.5% penicillin-streptomycin, and 0.25% amphotericin B (all supplied by Merck).

#### 3.5.2. Evaluation of EGFR Protein Levels

The EGFR protein levels were evaluated by Western blot analysis using total cellular extracts. Briefly, 1.0 × 10^6^ cells were seeded in cell culture flasks and allowed to grow for 24 h. Then cells were lysed with a buffer containing NaCl (120 mM), NaF (25 mM), EDTA (5 mM), EGTA (6 mM), sodium pyrophosphate (25 mM in TBS 20 mM, pH 7.4), PMSF (2 mM), Na_3_VO_4_ (1 mM), phenylarsine oxide (1 mM), 1% *v*/*v* NP-40 and 10% *v*/*v* Protease Inhibitor Cocktail. Protein concentration in cellular lysates was quantified using the bicinchoninic acid (BCA) assay. 30 μg of proteins per sample were then loaded onto a polyacrylamide gel (13%), and SDS-PAGE was performed. Proteins were transferred onto Hybond-P (Millipore, Milan, Italy) membranes to perform Western blot analysis using mouse monoclonal EGFR Antibody C-2. Bands are visualized by G-box (Syngene, Chemi-XT4, Fisher Scientific, Milan, Italy) using peroxidase-conjugated secondary anti-mouse antibodies (Santa Cruz Biotechnology, Inc., Milan, Italy) and Westar Supernova substrate (Cyanagen, Bologna, Italy). Equal loading of samples was checked by incubating the membrane with a mouse monoclonal anti-actin antibody (Santa Cruz Biotechnology, Inc., Milan, Italy).

#### 3.5.3. Intracellular Uptake and Localization

To assess photosensitizer (PS) uptake, flow cytometric analysis was performed by exploiting the intrinsic fluorescence of the compounds. Briefly, cells were seeded in 12-well plates at a density of 7.0 × 10^4^ cells per well and incubated with the compounds at a concentration of 100 nM for 24 h. The photoactivation step was omitted in this experimental setting. Following incubation, cells were harvested by trypsinization, washed with ice-cold PBS, resuspended in PBS, and analyzed using a FACScalibur flow cytometer (Becton Dickinson, Milan, Italy) equipped with a 15 mW, 488 nm air-cooled argon laser. Data acquisition and analysis were conducted using CellQuest Pro software version 5.1 (Becton Dickinson, Milan, Italy). Cellular uptake was quantified in arbitrary units based on median fluorescence intensity (MFI), by collecting PS fluorescence through a 575 nm band-pass filter.

To evaluate the contribution of enzymatic processes, specifically EGFR-dependent uptake, to overall PS internalization, a parallel set of cells was incubated in a 12-well plate maintained at 4 °C following treatment with PSs. At this temperature, enzymatic activity is effectively inhibited, preventing energy-dependent transport processes. Thus, PS uptake under these conditions reflects passive diffusion only, in the absence of receptor- or transporter-mediated mechanisms.

To investigate subcellular localization, cells were seeded onto glass coverslips at a density of 5 × 10^4^ cells/well and cultured for 48 h. Cells were then treated with 25 μM of each PS in combination with either 100 nM MitoView^®^ (mitochondrial marker, Merk, Milan, Italy) or LysoView^®^ (lysosomal marker, Merk, Milan, Italy). After 24 h of incubation, cells attached to the coverslips were washed three times with PBS and fixed with 3% paraformaldehyde in water (pH 7.4) for at least 10 min, followed by three additional PBS washes. Coverslips were then mounted onto microscope slides for imaging. Fluorescence images were acquired using the Leica Stellaris 5 confocal microscope (Leica Microsystems, Milan, Italy).

#### 3.5.4. Photodynamic Effects

The photodynamic effect of conjugates **6**–**8** on cell viability, compared to the reference compound **2**, was evaluated using the MTT ([3-(4,5-dimethylthiazol-2-yl)-2,5-diphenyltetrazolium bromide]) assay. Briefly, 3.5 × 10^3^ cells per well were seeded in 96-well plates and incubated for 48 h to allow for adherence and growth. Following this incubation period, cells were treated with increasing concentrations of the test compounds (ranging from 1 to 1000 nM), prepared in complete culture medium. All compound dilutions were prepared to ensure a final DMSO concentration of 0.1% (*v*/*v*), which is considered non-toxic. Control samples included cells cultured in complete medium alone, as well as a vehicle control treated with 0.1% DMSO in the absence of any compound (PS treatment was omitted). 24 h after treatment with PS, the medium was replaced with 1X PBS. Photoactivation was performed using a 500 W white tungsten halogen lamp, delivering an irradiance of 22 mW/cm^2^, corresponding to a total fluence of 158 J/cm^2^ over a 2 h irradiation period. To prevent thermal damage from the light source, a cooling system was employed by placing a circulating water filter between the lamp and the irradiation area to dissipate excess heat. Following irradiation, the PBS was replaced with fresh, PS-free culture medium, and cells were incubated at 37 °C. 24 h later, MTT was added to each well at a final concentration of 0.4 mg/mL, and cells were incubated for 3 h at 37 °C to allow the MTT metabolization in viable cells. The resulting formazan crystals were then dissolved in DMSO. Optical density was measured at 590 nm using an Infinite^®^ 200 PRO plate reader (Tecan Life Sciences, Switzerland), and the resulting data were analyzed by non-linear regression using GraphPad PRISM 9.2.0 software (GraphPad Software Inc., San Diego, CA, USA), and IC_50_ values were extrapolated from these curves.

The potential intrinsic cytotoxicity of the compounds was assessed under identical experimental conditions, except that the photoactivation step was omitted. In these experiments, cells were treated with compound concentrations up to ten times higher than the maximum concentration used in the photoactivation assay.

#### 3.5.5. Intracellular ROS Generation

Intracellular ROS generation was assessed using the fluorogenic probe 2′,7′-dichlorodihydrofluorescein diacetate (DCFH-DA; Invitrogen, Molecular Probes). Once internalized, DCFH-DA is hydrolyzed by intracellular esterases to form non-fluorescent dichlorodihydrofluorescein (DCFH), which ROS subsequently oxidizes to the highly fluorescent dichlorofluorescein (DCF). MDA-MB453 and MDA-MB231 cells were seeded in black 96-well plates and treated with the respective PSs at their IC_50_ concentrations for 24 h. Cells were then irradiated in the presence of 10 μM DCFH-DA. To quantify ROS production, fluorescence intensity was measured using a fluorescence microscope (excitation: 488 nm; emission: 520 nm). Quantitative analysis of fluorescence intensity, expressed in arbitrary fluorescence units, was performed using ImageJ software, version 1.53e, and normalized to untreated control samples.

#### 3.5.6. Cell Death Induction Evaluation

The ability of porphyrin **2** and its derivatives to induce cell death, either via apoptosis or necrosis, was assessed using flow cytometric analysis. MDA-MB231 and MDA-MB453 cells were seeded in 12-well plates (7.0 × 10^4^ cells per well) and allowed to grow for 48 h. Cells were then treated for 24 h with equitoxic concentrations of the PSs, corresponding to their respective IC_50_ values, irradiated for 2 h in PS-free PBS, and incubated for 24 h in fresh PS-free culture medium.

To evaluate the percentage of apoptotic cells, the cells were detached, washed twice in PBS, and fixed in 70% EtOH at −20 °C for at least 30 min. After a further wash in PBS, the cells were resuspended in a PBS solution containing propidium iodide (PI) (50 µg/mL) and RNase A (30 U/mL). All samples were analyzed using the FACSCalibur with the CellQuest PRO software. The fluorescence emission of PI was collected through a 575 nm band-pass filter, and the percentage of apoptotic cells in each sample was determined based on the sub-G1 peaks detected in the single-parameter histograms acquired in log mode.

For the evaluation of necrotic cells, the fixation step was omitted.

The potential induction of autophagy by the studied compounds was assessed by measuring LC3-II protein levels through Western blot analysis. MDA-MB231 and MDA-MB453 cells were seeded in cell culture flasks (1.0 × 10^6^ cells) and allowed to grow for 48 h. The cells were then treated with the compounds at their respective IC_50_ concentrations for 24 h, followed by 2 h of irradiation in 1X PBS. After irradiation, the cells were incubated for an additional 24 h in PBS-free medium. Western blot analysis was then performed as described in Section 3.5.2, using a rabbit polyclonal antibody against LC3-II (Merk, Milan Italy).

#### 3.5.7. Scratch Wound Healing Assay

The Scratch Wound Healing assay was performed to evaluate the potential effects of porphyrin 2 and its derivatives on the migration of MDA-MB453 and MDA-MB231 cells. Cells were seeded (1.5 × 10^5^ cells/well) on a 6-well plate, allowed to grow for 48 h to reach confluence, and treated with subtoxic concentrations of the compounds approximately corresponding with the respective IC_15_. After 24 h, a scratch was performed in each well using a pipette tip. Cells were then placed in 1X PBS, irradiated for 2 h, and placed in drug-free medium for 24 h.

Images of the scratch wound were taken immediately after scratch formation (t_0_) and after 24 h using a camera integrated with an Olympus IX81 microscope (Olympus, Milan, Italy). The percentage of the remaining open wound area was quantified using TScratch 1.1.2 software.

#### 3.5.8. Statistical Analysis

Statistical analysis of all biological data was performed using one- or two-way ANOVA, with Bonferroni’s test for multiple comparisons, using GraphPad PRISM 8.43 software.

## 4. Conclusions

In the present study, novel peptide–porphyrin conjugates targeting EGFR were successfully synthesized using an original and efficient method. The conjugation was performed under mild conditions to preserve the structural integrity and functionality of the peptide moiety, and the process yielded the final products in satisfactory amounts. Cellular uptake studies demonstrated that the EGFR-overexpressing MDA-MB231 cell line preferentially internalized the conjugates, while significantly lower uptake was observed in the EGFR-negative MDA-MB453 cells. These findings support our initial hypothesis that the designed peptides can specifically target EGFR-expressing cells. Moreover, the peptide–porphyrin conjugates exhibited enhanced photodynamic activity in TNBC cell lines compared to the nitrated porphyrin used as a reference compound, likely due to improved cellular internalization. Additionally, the two cell lines displayed different extents of PDT-induced ROS production, as well as varying degrees of apoptotic, necrotic, and autophagic cell death, highlighting cell-specific responses to the treatment.

In the present study, relatively high light doses were applied to ensure sufficient excitation of the photosensitizer under the specific experimental conditions. These doses, however, may indicate an inefficient use of light energy and differ from those typically used in vivo or with laser-based protocols. Future optimization, including the adoption of light sources with narrower wavelength profiles and higher photon flux, may improve the efficiency and reproducibility of photodynamic responses.

A notable and clinically relevant finding was the strong antimigratory effect of EGFR-targeted PDT in the MDA-MB231 cell line. This result highlights the potential of EGFR-targeted PDT to specifically inhibit the migratory ability of aggressive breast cancer cells that overexpress EGFR, which is closely linked to metastatic progression. The lack of a significant antimigratory effect in the non-EGFR-expressing MDA-MB453 cells, along with the absence of activity without photoactivation, further emphasized the high selectivity and precise control of this therapeutic method. Such specificity is particularly beneficial for clinical use, as it may minimize off-target effects and reduce damage to surrounding healthy tissues.

Future studies will focus on extending these findings to other metastatic traits, such as invasion and extracellular matrix degradation, and validating therapeutic efficacy in relevant in vivo models. Mechanistic investigations into intracellular trafficking and PDT-induced signaling pathways will provide further insight into the observed antimigratory effects. We acknowledge that in vivo validation and evaluation of EGFR heterogeneity are essential for clinical translation; these will also be addressed in follow-up work assessing (i) therapeutic efficacy and safety in animal models and (ii) the influence of intratumoral EGFR heterogeneity on treatment outcomes.

## Data Availability

The original contributions presented in this study are included in the article/Supplementary Materials. Further inquiries can be directed to the corresponding author.

## Supplementary Materials

The following supporting information can be downloaded at: https://www.mdpi.com/article/10.3390/molecules30173533/s1, Figure S1: Overview of peptide sequences designed using a rational design approach; Figure S2: LC–MS analysis, showing the formation of multiple by-products, suggestive of peptide backbone degradation beyond 30 h; Figure S3: Structures of porphyrin conjugates **6**–**8**; Figure S4: EGFR protein levels in MDA-MB453 and MDA-MB231 whole cell lysates; Figure S5: Assessment of mitochondrial localization of conjugate **7** in MDA-MB-453 and MDA-MB-231 cells; Figure S6: Survival fraction obtained in MCF7, MDA-MB453, and MDA-MB231 cell lines following treatment with the four compounds tested (**2**, **6**–**8**) at a concentration corresponding to ten times the maximum concentration used during the PDT experiments (10 mM). Mean ± SD of 5 independent experiments; Figure S7: Uncropped images of LC3-II Western blot experiments; Figure S8: Scratch Wound Healing assay performed on MDA-MB453 and MDA-MB231 cell lines following 24 h treatment with subtoxic concentrations of porphyrin **2** and its derivatives, and PDT. Representative images.
