# Supplementary material for: EGFR-Targeted Photodynamic Treatment of Triple Negative Breast Cancer Cell Lines Using Porphyrin–Peptide Conjugates: Synthesis and Mechanistic Insight
Source: Molecules. 2025 Aug 29;30(17):3533. doi: 10.3390/molecules30173533 (PMC12429880; doi:10.3390/molecules30173533)
Supplement: Supplementary file 1 [file molecules-30-03533-s001.zip › molecules-3813124-supplementary.pdf]

Supplementary materials

Targeted photodynamic therapy of Triple Negative Breast Cancer using EGFR-directed Porphyrin-peptide conjugates: synthesis and mechanistic insight.

Miryam Chiara Malacarne[1], Federica Randisi[1], Emanuela Marras[1], Stefano Giovannardi[1, #], Paolo Dognini[2], Alan Simm[3], Francesca Giuntini[2], Marzia Bruna Gariboldi[1, *], Enrico Caruso[1]

[1]Department of Biotechnology and Life Sciences (DBSV), University of Insubria, via J.H. Dunant 3, 21100 Varese (Italy); mc.malacarne1@uninsubria.it; frandisi1@studenti.uninsubria.it; emanuela.marras@uninsubria.it; stefano.giovannardi@uninsubria.it; marzia.gariboldi@uninsubria.it; enrico.caruso@uninsubria.it

[2]School of Pharmacy and Biomolecular Sciences, Byrom Street Campus, Liverpool John Moores University, Liverpool L3 3AF (United Kingdom); paolo.dognini@ambiopharm.com; F.Giuntini@ljmu.ac.uk;

[3] Faculty of Sciences, Byrom Street Campus, Liverpool John Moores University, Liverpool L3 3AF (United Kingdom); A.M.Simm@ljmu.ac.uk;

Centre for Neuroscience, University of Insubria, Varese, Italy.

*Correspondence: marzia.gariboldi@uninsubria.it (MBG).

Figure S1. Overview of peptide sequences designed using a rational design approach.
Figure S2. LC–MS analysis, showing the formation of multiple by-products, suggestive of peptide backbone degradation beyond 30 hours.
Figure S3. Structures of porphyrin conjugates **6-8**.
Figure S4. EGFR protein levels in MDA-MB453 and MDA-MB231 whole cell lysates.
Figure S5. Assessment of mitochondrial localization of conjugate **7** in MDA-MB-453 and MDA-MB-231 cells.
Figure S6: Survival fraction obtained in MCF7, MDA-MB453, and MDA-MB231 cell lines following treatment with the four compounds tested (**2**, **6**- **8**) at a concentration corresponding to ten times the maximum concentration used during the PDT experiments (10 μM). Mean ± SD of 5 independent experiments.
Figure S7. Uncropped images of LC3-II western blot experiments
Figure S8. Scratch Wound Healing assay performed on MDA-MB453 and MDA-MB231 cell lines following 24h treatment with subtoxic concentrations of porphyrin **2** and its derivatives and PDT. Representative images.

	Sequence
**Peptide 1**	CHWNGYTPENVI
**Peptide 2**	CHWDGYTPENVI
**Peptide 3**	CHWQGYTPENVI
**Peptide 4**	CHWYGYTPENVI
**Peptide 5**	CHWYGYTPQNVI
**Peptide 6**	CHWYGYSPENVI
**Peptide 7**	CHWYGYQPENVI
**Peptide 8**	CHWYGYTPENLI
**Peptide 9**	CFWYGYTPENVI
**Peptide 10**	CWWYGYTPENVI
**Peptide 11**	CGWYGYTPENVI
**Peptide 12**	CYWYGYTPENVI
**Peptide 13**	CKWYGYTPENVI
**Peptide 14**	CHWYAYTPENVI
**Peptide 15**	CHWNGYTPENVI

Figure S1. Overview of peptide sequences designed using a rational design approach.

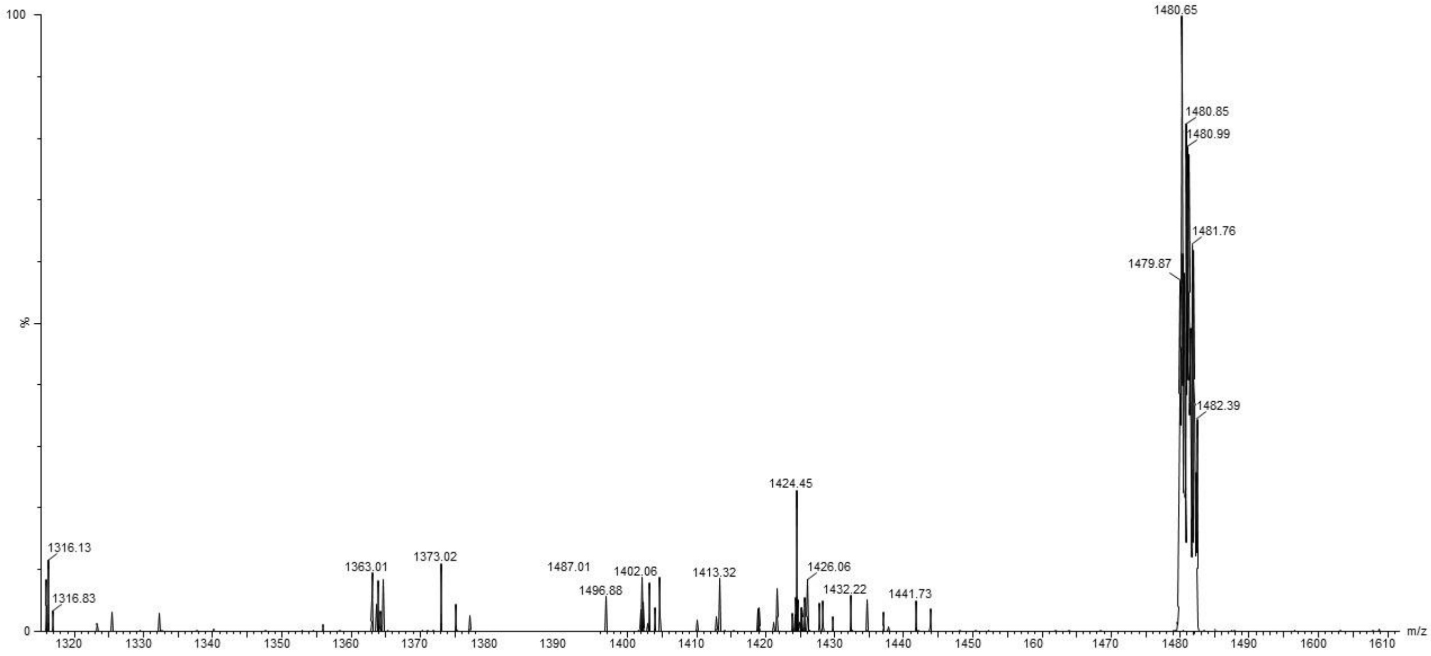

Figure S2. LC–MS analysis, showing the formation of multiple by-products, suggestive of peptide backbone degradation beyond 30 hours.

6

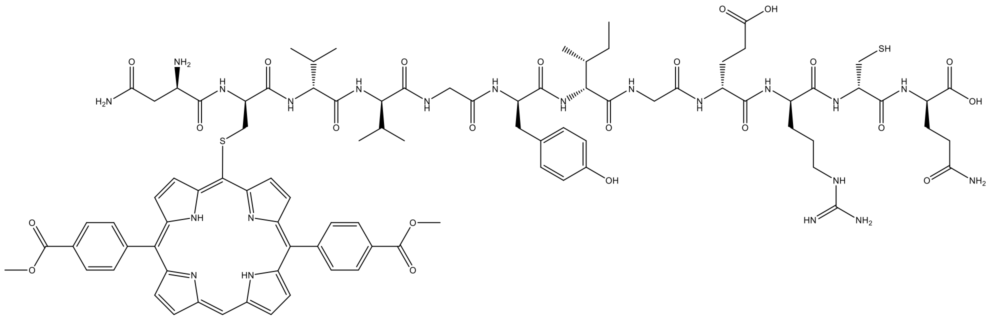

7

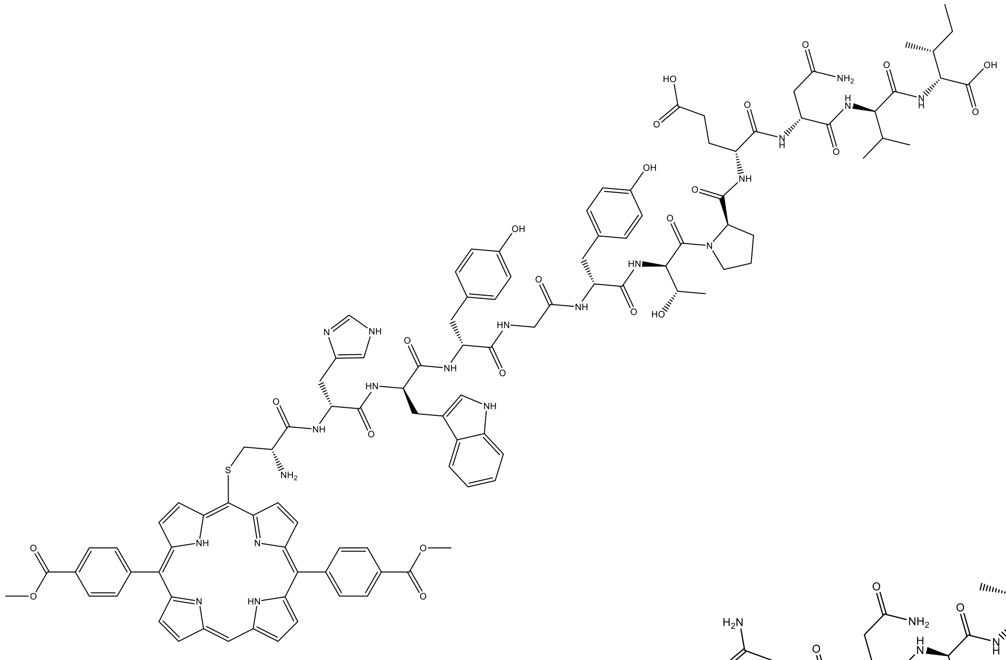

8

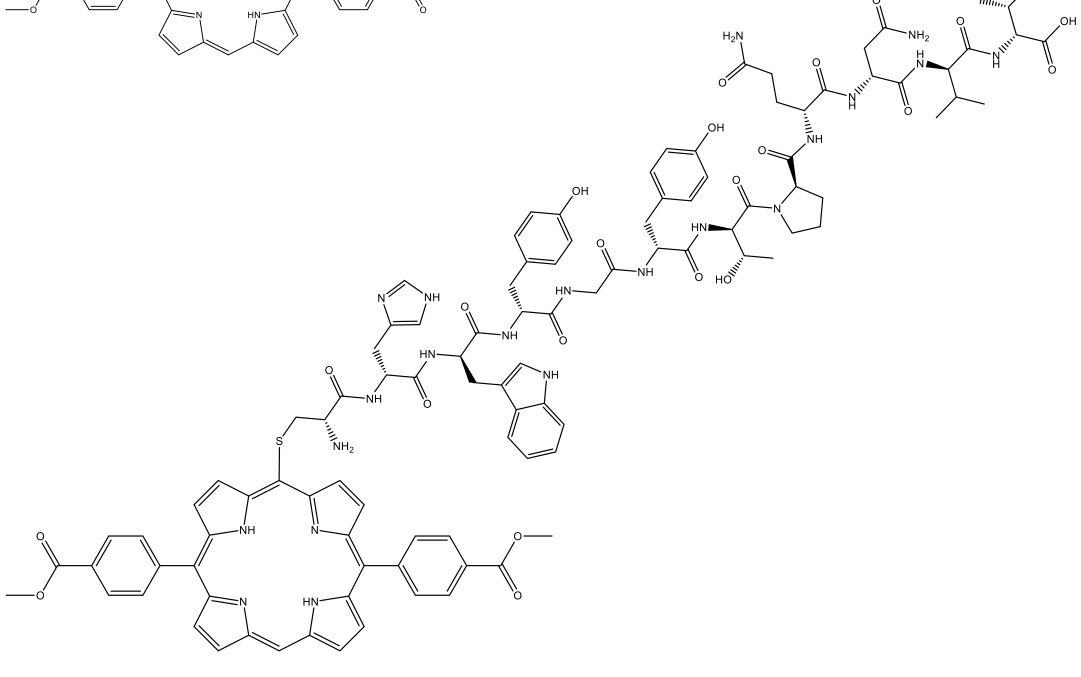

Figure S3. Structures of porphyrin conjugates **6**-**8**.

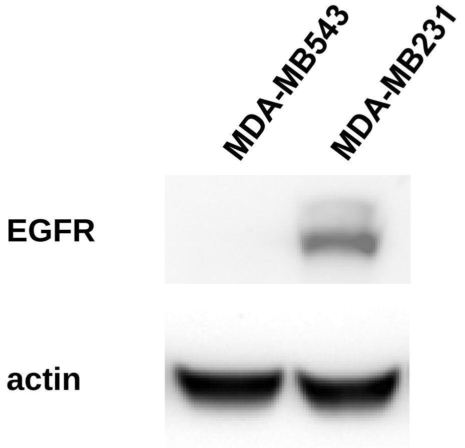

Figure S4. EGFR protein levels in MDA-MB453 and MDA-MB231 whole cell lysates.

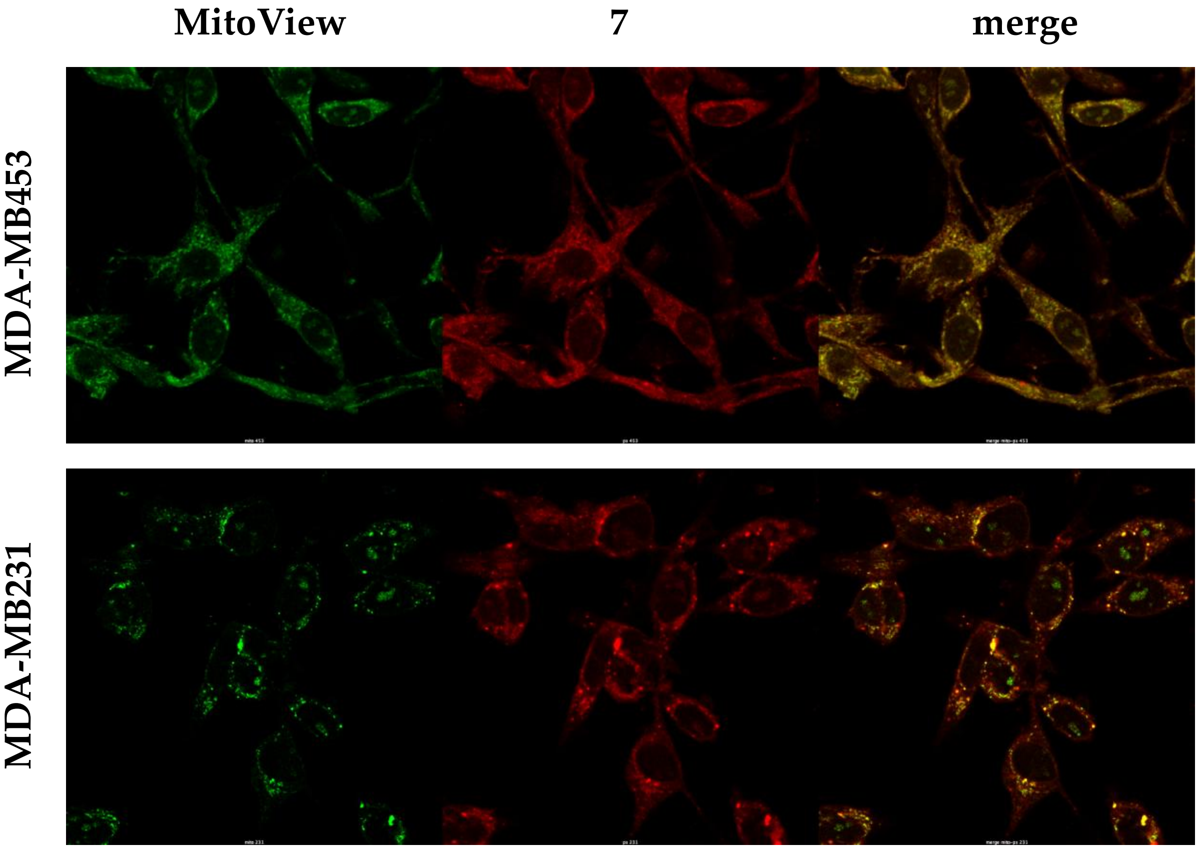

Figure S5. Assessment of mitochondrial localization of conjugate **7** in MDA-MB-453 and MDA-MB-231 cells.

	MCF7	MDA-MB453	MDA-MB231
**2**	89.7 ± 1.16	90.8 ± 0.66	91.2 ± 1.26
**6**	96.3 ± 1.15	95.7 ± 0.82	98.6 ± 0.56
**7**	97.3 ± 0.73	96.8 ± 0.86	96.8 ± 1.35
**8**	95.2 ± 0.75	94.7 ± 0.97	95.9 ± 0.70

Figure S6. Survival fraction obtained in MCF7, MDA-MB453, and MDA-MB231 cell lines following treatment with the four compounds tested (2, **6**- **8**) at a concentration corresponding to ten times the maximum concentration used during the PDT experiments (10 μM). Mean ± SD of 5 independent experiments.

MDA-MB453

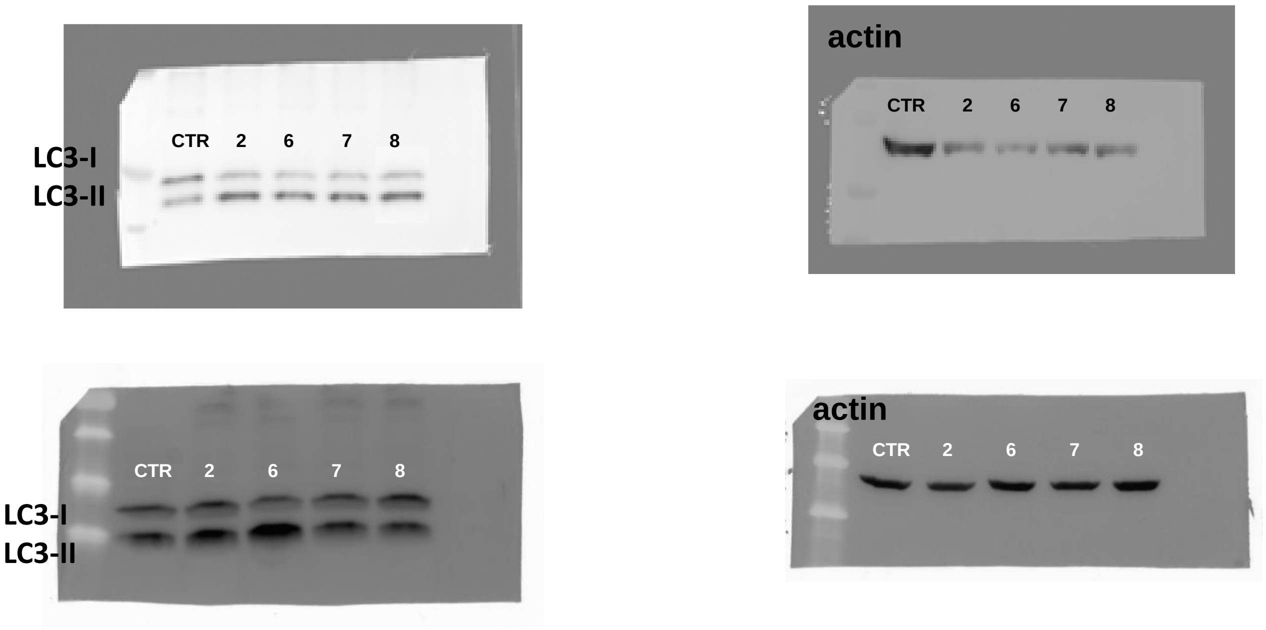

MDA-MB231

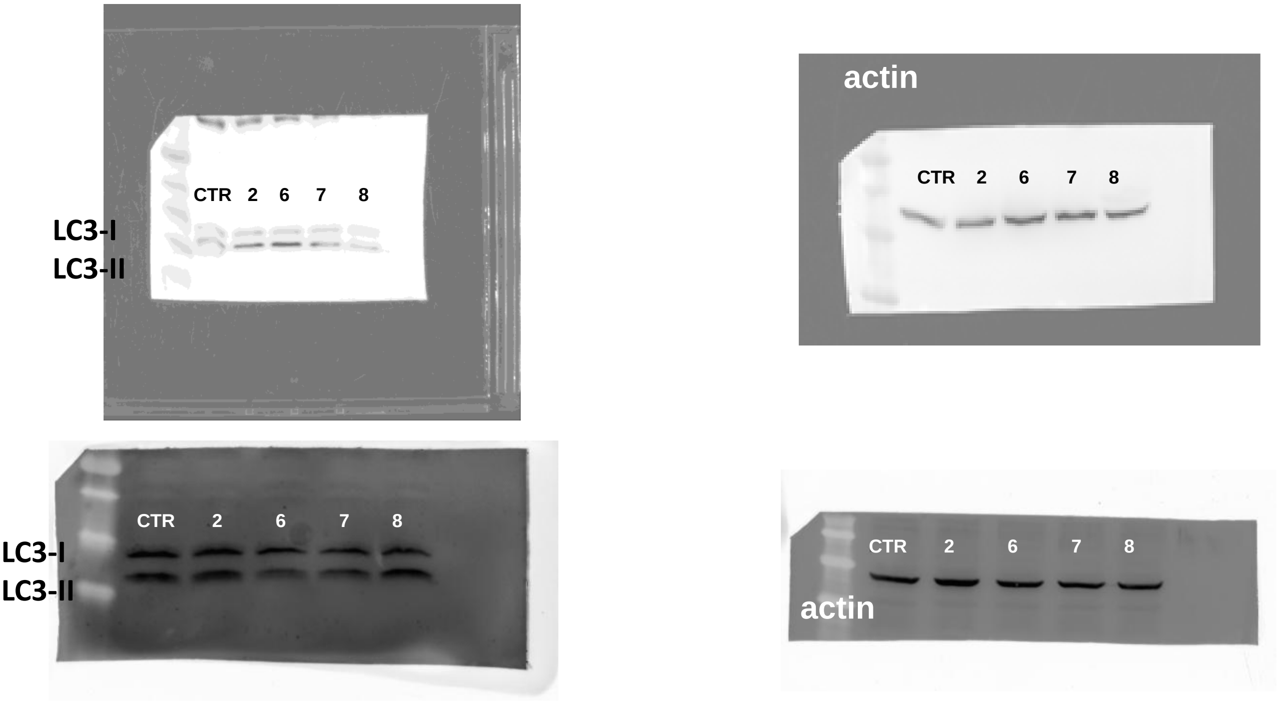

Figure S7. Uncropped images of LC3-II western blot experiments.

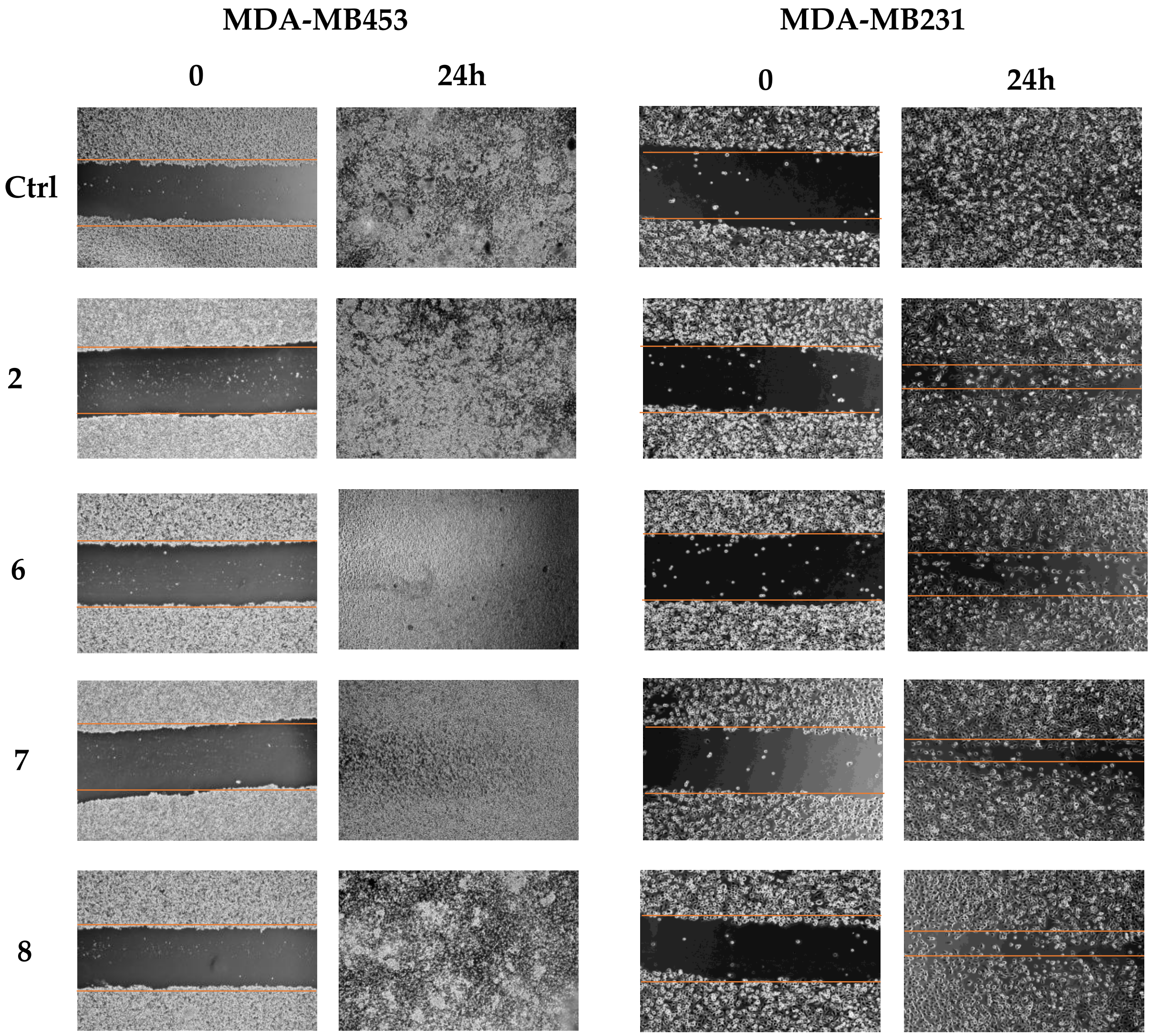

Figure S8. Scratch Wound Healing assay performed on MDA-MB453 and MDA-MB231 cell lines following 24h treatment with subtoxic concentrations of porphyrin **2** and its derivatives and PDT. Representative images.
